# Child health guidelines in the era of sustainable development goals

**DOI:** 10.1136/bmj.k3151

**Published:** 2018-07-30

**Authors:** Jonathon L Simon, Bernadette Daelmans, Cynthia Boschi-Pinto, Samira Aboubaker, Wilson Were

**Affiliations:** 1 World Health Organization, Geneva, Switzerland

## Abstract

Child health guidelines must broaden in scope beyond under 5 mortality to facilitate improvements in child health and development necessary to meet the 2030 sustainable development goals, say **Jonathon L Simon and colleagues**

As the United Nations coordinating authority for international health, the World Health Organization has a vital role in producing scientifically valid and up to date global guidance. It is a challenge to keep the guidelines relevant and evidence based amid changing demography and disease epidemiology and expansion of scientific information and new priorities.

The Integrated Management of Childhood Illness (IMCI) strategy was launched in 1995. It aimed to collate technical guidance related to management of the leading causes of childhood mortality in a holistic and child centred way. These guidelines were developed through review of evidence and based on expert consensus recommendations. Since 2007, a systematic review of evidence has been followed using the GRADE (Grading of Recommendations Assessment, Development and Evaluation) approach, managed by an independent guideline review committee.^1^

Since the IMCI strategy was launched most countries have made substantial progress in reducing mortality among children under 5 years old. Overall, global mortality among under 5s has declined by an estimated 56% since 1990.^2^ During this period, the global child health environment has changed markedly with the advent of sustainable development goals and epidemiological and demographic transitions. WHO is undertaking a comprehensive review of the child health guidelines to make them more appropriate for the needs of different countries and situations.^3^

Here, we discuss the changes needed based on the changing epidemiology and the analytical background papers and recommendations from the 2016 strategic review of IMCI and the results of the IMCI global implementation survey.^4^ In addition, we draw on the strategic direction provided by the Global Strategy for Women’s, Children’s and Adolescents’ Health.^5^ The analytical approach and overall recommendations are described in the overview paper of this collection.^6^

## History and evolution of IMCI guidelines

At its inception in the mid-1990s, IMCI focused on countries where the mortality rate in the under 5s was higher than 40 per 1000 live births.^7^ In 1995, these countries comprised half of the 194 WHO member states. The mortality rate in under 5s fell to 42% in the year 2000 and to 30% in 2015.^8^ In addition, the main causes of child death have been changing. In 2000, more than half of the 9.8 million deaths in the under 5s were due to infectious diseases such as pneumonia, diarrhoea, malaria, and measles. By 2015 this proportion had declined to 43% for many reasons, including economic growth, nutritional improvements, and health interventions.

Pneumonia, newborn sepsis, and diarrhoea remain among the top causes of death in young children. However, sequelae of newborn asphyxia and prematurity, congenital anomalies, non-communicable diseases, and injuries are becoming increasingly common causes of morbidity and mortality in countries where under-5 mortality has declined.^7^ When the mortality rate in under 5s declines from 55 per 1000 live births to 20 per 1000 live births, the relative contribution of congenital anomalies, non-communicable diseases, and injuries is likely to increase from 12% to 34% of all deaths in this group. Meanwhile, the contribution of infectious diseases is likely to decline from 53% to 24% of all deaths in the under 5s.^9^ In the next 15 years such changes are likely to occur in the 58 countries where current mortality in children under 5 is at least 40 per 1000 live births as they move towards the sustainable development goal of reducing their under-5 mortality rate to ≤25 per 1000 childbirths by 2030.

The initial IMCI case management guidelines, tools, and related training materials concentrated on the main conditions causing childhood mortality. Over time, countries have included other conditions and have adapted and updated the IMCI guidelines according to their regional and national epidemiological situation. The recent IMCI global implementation survey^4^ ^10^ reported that about 100 countries had adapted and used the IMCI. Since the last major global technical update in 2014, more than 80% of the countries had updated the national chart booklet which serves as the core training and job aid. Over half updated their national treatment guidelines for hospital care based on the 2013 version of the *WHO pocket book of hospital care for children*.^11^

Adaptations were widespread and country specific (table 1). Responding to the increased relative burden of newborn mortality, many countries modified their national IMCI algorithm to include care of the sick newborn in the first week of life. The common conditions added included HIV, and sore throat, skin conditions, and tuberculosis in all except the European region, and dengue haemorrhagic fever (mainly in the Americas, South East Asia, and Western Pacific regions).

**Table 1:** Proportion of countries by region that adapted the Integrated Management of Childhood Illness guidelines for other specific diseases and conditions

Disease	African **(%)**	Americas (%)	Eastern Mediterranean (%)	European (%)	South East Asia (%)	Western Pacific (%)
Dengue	2	73	15	0	67	56
Tuberculosis	44	47	15	0	44	33
HIV	93	67	15	33	44	33
Skin condition	46	47	23	0	67	67
Sore throat	39	73	77	100	56	78
Jaundice	73	73	92	83	78	78
Others	39	73	31	33	56	22

It is a challenge to keep the IMCI guidelines up to date with the changing clinical epidemiology of disease, but they have contributed to reductions in deaths due to diarrhoea, pneumonia, and malaria over the past 20 years.^12^ Although up to date guidelines are important, they are not by themselves sufficient to establish universal health coverage with quality services. Moving beyond “survive” to meet the objectives of the “thrive” and “transform” components of the Global Strategy for Women’s, Children’s and Adolescents’ Health is imperative so that all children, irrespective of where they are born, attain similar levels of health. The goal is to end preventable child mortality and ensure that every child’s health, growth, and development are sufficient for them to meet their full potential.

## Rationale for re-examining child health guidance

One of the key recommendations of the child health strategic review was a call for WHO and Unicef to bring together existing guidance packages for newborns and children under 5 into one set of flexible, adaptable, and user friendly tools. The review also called for an approach that dealt with child health and development during the first 18 years of life. The life course approach, considers “childhood” as defined by the UN Declaration on the Rights of the Child (ages 0-18 years) and calls for a continuum of support and care where inputs at each age become the basis for improving the health and wellbeing at each subsequent age. This call for the redesign of the child health guidance within the context of this life course approach and the sustainable development goals is based on six identified factors that create changes and require a continued focus on child survival while also broadening the concept of global child health.

Firstly, the change from millennium development goals to sustainable development goals has extended the programme. In addition to reduction of the mortality of young children, it now examines the social determinants of ill health and improved wellbeing. The Global Strategy for Women’s, Children’s and Adolescents’ Health emphasises the importance of an approach to ensure that children aged 0-18 years can survive and thrive.^3^ Thus the focus has expanded beyond preventing mortality in the under 5s. The broader agenda requires, among other things, guidelines that deal with early childhood development, environmental health, adolescent mental health, and prevention and management of nutritional disorders (under- and overnutrition) and their links to non-communicable diseases. It also challenges us to meet the largely ignored needs of children aged 5-9 years. This would bridge the gap between child health programming for children under 5 and the emerging adolescent health plans. 

Secondly, changes in the timing and causes of child deaths suggest that greater attention should be paid to the first year of life. Focus on the first day, week, and month of life is key as this is when most deaths occur. Congenital abnormalities are increasingly dominant in settings where neonatal mortality from causes such as asphyxia, prematurity, and neonatal sepsis has been reduced. Identifying, testing, and communicating effective and feasible interventions to save lives and foster wellbeing of newborn infants at the differing levels of the health system requires robust research.

Thirdly, the geographical distribution of deaths has changed markedly over the past 20 years. The largest demographic change in history is occurring as the world’s population is now predominantly located in urban and peri-urban areas. Not only does this change transmission dynamics and response to disease outbreaks (for example, the impact of the Ebola epidemic in west Africa), but it also changes health seeking behaviours as problems of geographical access are supplanted by economic problems. Urbanisation leads to rapid growth in use of the profit and not for profit private sectors for routine health services. In addition, increased recognition of the damage imposed by conflict and other humanitarian emergencies on the health of women and children drives us to improve our understanding of the locational problems associated with death and morbidity. These concerns are now poorly covered and attempts are under way to fill this gap.

Fourthly, advances in clinical sciences and biomedical technology create opportunities to improve existing guidance. The introduction of new vaccines and antibiotics has changed the epidemiology, prevention, and treatment of several conditions. Old and new conditions and technologies would benefit from an updated review to ensure that they reflect the most recent scientific evidence. For example, research is under way to strengthen clinical recommendations for identification of children with signs of pneumonia who are likely to benefit from antibiotics. A continuous and careful review of new evidence, timely expert consultation, and a global guidelines process that facilitates rapid updates to current recommendations is therefore required. This will ensure that the WHO/Unicef guidance is up to date. Work into altering strategies for dissemination of WHO technical information is continuing. The use of digital platforms will help and facilitate more rapid updates of technical information and enable the democratisation of information. Some suggest that materials should be available to multiple audiences (including simplified versions for end users) as well as Ministry of Health policy and planning officers.

Fifthly, changes due to complexity and variability in different areas are increasingly recognised as critical for effective programming, irrespective of the level of socioeconomic development. A consistent finding from the global survey was that epidemiological and health systems differ markedly across regions, countries, and within countries. Although intercountry differences are well recognised, intracountry variability is equally important. High risk and marginalised populations exist within every country and the commitment to increasing equity and universal health coverage requires that guidance reaches the most vulnerable populations. This forms part of the rationale for customising technical messages to better reach end users and service providers of marginalised populations to reduce information asymmetries.

Across regions, countries have very different levels of health systems development and human resource capacity. In addition, with increasing awareness of the level of mortality and morbidity in children in conflict and emergency humanitarian settings, the need to produce materials to deal with the requirements of such children is immediate. Clearly, universal guidance and programming cannot hope to deal with the needs of these widely varying situations. WHO will seek to produce more guidance documents that are specific to the varying environments.

Finally, the broad sustainable development goals and changes requiring guidance, such as urbanisation, climate change, environmental degradation, and increasing antimicrobial resistance, will need to be given priority. The broader agenda of child health and development thus requires integrated guidelines at each stage of life to consider health promotion, disease prevention, and the quality care and treatment of neonates, young children, and adolescents.

## Guideline and guidance priorities

Developing WHO technical guidelines to meet the sustainable development goals is challenging given the breadth of problems reflected in the 17 goals, with a broad definition of health being a core component of many of them. WHO and Unicef envisaged a child health redesign project to deal with these challenges through consultation with policy makers, programme managers, scientists, implementation partners, and civil society in all regions. This process has identified priority areas on which WHO and Unicef will focus. There is general agreement that the emphasis should expand beyond survival of children under 5. The changing patterns of mortality require a greater focus on interventions to reduce mortality in the first week, month, and year of life. Expansion of early child development activities in the first 3 years may help to ensure that children have the greatest chance to thrive and meet their potential. A strong focus on nutrition across all age groups, considering both undernutrition and overnutrition, is a priority. The largest gap to fill in guidance may be for children aged 5-9 years, a neglected group. For these children accidents and injuries are increasingly important causes of death, requiring the scope of guidance to be broadened. Furthermore, we acknowledge that integrating medical, health promotion, mental health, and social/behaviour interventions across the age groups and differing health settings may be a complex challenge.

As a consequence of this expanded vision, three priority areas for guidelines and guidance work have been identified: updating current guidelines using the life course approach; identifying and filling guideline gaps, starting with children aged 5-9 and thrive interventions; and identifying innovative ways of making guidelines flexible and adaptable to local priorities. This work is large, broad, complex, and important. It will require WHO and Unicef, working with multiple stakeholders, to commit to many years of effort to listen, learn, and adapt as we change the way in which guidelines are devised, developed, and disseminated.

## Summary and conclusions

The sustainable development goals have placed additional responsibilities upon WHO and Unicef to ensure that guidance meets the broader needs and deals with the newer questions that countries face as they respond to their global commitments. For child health, this means promulgation of a new vision, in which child health and development are dealt with in an innovative, evidence based, and balanced way. A major initiative to revise and update existing child health guidelines and create the new guidance necessary for the expanded goals is urgently needed.

The transition from the millennium development goals to the sustainable development goals, and WHO’s continuing commitment to the health of women, children, and adolescents provides an opportunity to re-examine child health programming. The emphasis on child survival that marked the past two decades was justifiable, but a move to consider child health in a holistic and child centred way is of utmost importance to deal with the root causes of poor health and development and reduce inequities. Countries ascribe great importance to WHO guidance. Thus, the need to adopt state of the art thinking and science is both a huge responsibility and an opportunity for WHO. This is an exciting opportunity to refresh the world’s child health knowledge and, more importantly, use this information to accelerate the improvements in child health and development required to meet the 2030 sustainable development goals.

**Key messages**

•Many epidemiological and demographic changes affecting child health have occurred since the launch of the Integrated Management of Childhood Illness strategy
The sustainable development goals demand the focus to be widened beyond mortality among children under 5 years
Guidelines need to be updated to take account of these changes as well as scientific advances
A more holistic view of child health is needed that includes environmental and social determinants

## Notes

See www.bmj.com/child-health for other articles in the series.

Contributors and sources: JLS conceptualised the commentary, wrote the initial draft with inputs from others and finalised the version, incorporating the contributions from the coauthors. BD, contributed ideas to the concept, helped to write the first draft, and commented and edited subsequent drafts. CB-P, SA, WW contributed ideas to the concept, and commented and edited subsequent drafts. CB-P provided most of the estimates of the changing mortality. All authors were members of the Coordinating Group of the Child Health (IMCI) Strategic Review.

Provenance and peer review: Commissioned; externally peer reviewed.

This article is part of a series based on findings from a global review of Integrated Management of Childhood Illness funded by the Bill and Melinda Gates Foundation. Open access fees were funded by the Health Systems Research Unit, South African Medical Research Council. *The BMJ* peer reviewed, edited, and made the decision to publish the article with no involvement from the Medical Research Council.
